# Book Reviews

**Published:** 1804-02-01

**Authors:** 


### CRITICAL ANALYSIS

### OF THE

### RECENT PUBLICATIONS

### ON THE

### DIFFERENT BRANCHES OF PHYSIC, SURGERY, AND MEDICAL PHILOSOPHY.

*Medical Ethics; or a Code of Institutes and Precepts, adapted to the Professional Conduct of Physicians and Surgeons; to which is added an Appendix, containing a Discourse on Hospital Duties; also Notes and Illustrations. By* THOMAS PERCIVAL, M. D. Manchester, 1803.

MUCH of this interesting work has already appeared before the Public in one form or other; but we have great pleasure in seeing the whole collected together, as forming a code of most excellent regulations for professional men, every clause of which exhibits that liberal and gentleman-like spirit which is always expected from, and so often adorns, the well educated British Physician.

The first chapter, the author observes in his preface, was composed in 1792, at the request of the physicians and surgeons of the Manchester Infirmary, and is the substance of the laws by which the practice of that institution (as far as relates to the mutual conduct of the medical attendants) has since been regulated.

The second chapter includes the more extended subject of professional conduct in private or general practice. Earnestly recommending a perusal of the whole, we shall select a few parts which are more peculiarly professional, or such as are intended to regulate conduct in cases to which the common laws of morality or good manners do not so immediately apply; for, after all, it is only these that are calculated to excite much interest from the medical reader. First, as to interference;

"*Officious Interference*, in a case under the charge of another, should be carefully avoided. No meddling inquiries should be made concerning the patient; no unnecessary hints given, relative to the nature or treatment of his disorder; nor any selfish conduct pursued, that may directly or indirectly tend to diminish the trust reposed in the physician or surgeon employed. Yet though the character of a professional busy-body, whether from thoughtlessness or craft, is highly reprehensible, there are occasions which not only justify but require a spirited interposition. When artful ignorance grossly imposes on credulity; when neglect puts to hazard an important life; or rashness threatens it with still more imminent danger; a medical neighbour, friend, or relative, apprized of such facts, will justly regard his interference as a duty. But he ought to be careful that the information, on which he acts, is well founded; that his motives are pure and honourable; and that his judgement of the measures pursued is built on experience and practical knowledge, not on speculative or theoretical differences of opinion. The particular circumstances of the case will suggest the most proper mode of conduct. In general, however, a personal and confidential application to the gentleman of the faculty concerned, should be the first step taken, and afterwards, if necessary, the transaction may be communicated to the patient or to his family."

*Professional Assistance.* — "Whenever a physician or surgeon *officiates* for another, who is sick or absent, during any considerable length of time, he should receive the fees accruing from such additional practice. But if this fraternal act be of short duration, it should be gratuitously performed; with an observance always of the utmost delicacy towards the interest and character of the professional gentleman, previously connected with the family."

*Fees.*—"Some general rule should be adopted by the faculty, in every town, relative to the *pecuniary acknowledgements* of their patients; and it should be deemed a point of honour to adhere to this rule rule with as much steadiness, as varying circumstances will admit. For it is obvious that an average fee, as suited to the general rank of patients, must be an inadequate gratuity from the rich, who often require attendance not absolutely necessary; and yet too large to be expected from that class of citizens, who would feel a reluctance in calling for assistance, without making some decent and satisfactory retribution."

*Quack Medicines.*—"The use of these should be discouraged by the faculty, as disgraceful to the profession, injurious to health, and often destructive even of life. Patients, however, under lingering disorders, are sometimes obstinately bent on having recourse to such as they see advertised, or hear recommended, with a boldness and confidence, which no intelligent physician dares to adopt with respect to the means that he prescribes. In these cases, some indulgence seems to be required to a credulity that is insurmountable. And the patient should neither incur the displeasure of the physician, nor be entirely deserted by him. He may be apprized of the fallacy of his expectations, whilst assured, at the same time, that diligent attention should be paid to the process of the experiment he is so unadvisedly making on himself, and the consequent mischiefs, if any, obviated as timely as possible. Certain active preparations, the nature, composition, and effects of which are well known, ought not to be proscribed as quack medicines."

The last is a subject of some difficulty, as the author himself appears to feel by the concluding sentence, which evades the general question, but does not decide it.

*Gratuitous Advice.*—"A wealthy physician should not give advice *gratis* to the affluent; because it is an injury to his professional brethren. The office of physician can never be supported but as a lucrative one; and it is defrauding, in some degree, the common funds for its support, when fees are dispensed with, which might justly be claimed." This is equally just and prudent.

Was it the author's knowledge of mankind that suggested to him the following clause?

"The commencement of that period of senescence, when it becomes incumbent on a physician to decline the offices of his profession, it is not easy to ascertain; and the decision on so nice a point must be left to the moral discretion of the individual. For, one grown old in the useful and honourable exercise of the healing art, may continue to enjoy, and justly to enjoy, the unabated confidence of the public. And whilst exempt, in a considerable degree, from the privations and infirmities of age, he is under indispensable obligations to apply his knowledge and experience in the most efficient way, to the benefit of mankind. For the possession of powers is a clear indication of the will of our Creator, concerning their practical direction. But in the ordinary course of nature, the bodily and mental vigour must be expected to decay progressively, though perhaps slowly, after the meridian of life is past. As age advances, therefore, a physician should, from time to time,

 scrutinize scrutinize impartially the state of his faculties; that he may determine, *bona fide*, the precise degree in which he is qualified to execute the active and multifarious offices of his profession. And whenever he becomes conscious that his memory presents to him, with faintness, those analogies, on which medical reasoning and the treatment of diseases are founded; that diffidence of the measures to be pursued perplexes his judgement; that from a deficiency in the acuteness of his senses, he finds himself less able to distinguish signs, or to prognosticate events; he should at once resolve, though others perceive not the changes which have taken place, to sacrifice every consideration of fame or fortune, and to retire from the engagements of business."

The conduct of physicians to apothecaries forms the subject of the third chapter, at the end of which he strongly recommends the establishment of benefit societies similar to those already established in Norfolk and London, for the most numerous class of professional men.

The fourth chapter relates to those cases in which medical men, as such, are concerned with the laws of their country, and with this terminates the juridical part of this collection.

Two additions are made, in the form of appendix; the first, a Discourse on Hospital Duties by the Rev. Thomas Bassnett Percival; and the second, consisting of Notes and Illustrations to the former part of the work, by the author. The most prominent of these, and one in which the author appears to speak with his whole heart, is a defence of the medical profession from the old and often repeated charge of scepticism in matters of religion.

Here we shall conclude our remarks on this candid and interesting treatise, abounding in humanity and good sense; but as a code for practical use, much too limited and general to apply to almost the only cases in which specific advice might be acceptable.

---

*Essays on the Diseases of Children, Essay II. on the Bowel Complaints more intimately connected with the Biliary Secretion, and particularly of the Atrophia Ablactatorum, or Weaning-Brash. By* John Cheyne, M. D.

The first Essay of this author, on Croup, we noticed in a former number of our Journal; in the present treatise Dr. C. describes some very important diseases connected with the biliary system, to which infants are liable; and which annually carry off a very large number of the young of the human species.

The first which the author mentions is Jaundice, which attacks infants a few days after birth, attended with languor, flatulence, and bilious urine, and appears at once with such decided symptoms as to indicate a probable organic derangement in the structure of the liver. It is always dangerous, and generally fatal. The author has not found any considerable light thrown on the immediate cause of the disease by dissection, and therefore supposes it with some some probability to depend on some preternatural thickening of the pori biliari. A slighter kind of jaundice is however described by several authors, which disappears in a few days.

A plate is given, exhibiting a dissection of a case of the former species of jaundice, in which the principal morbid appearance was a great enlargement of the liver to nearly twice its natural size.

But the most important disease considered in this Essay is what is commonly (in Scotland) called the *Weaning Brash*, and to which Dr. C. gives the appropriate term of *Atrophia Ablactatorum.* It is a morbid state of all the digestive functions of children, apparently produced by a want of the natural food, the mother's milk, and is well known to every practitioner, particularly in large towns, and to every nurse. How many hundreds of instances annually occur in this metropolis, of infants, apparently stout and healthy at birth, who continue to thrive perfectly well for several weeks, till the mother, confiding in the signs of robust health in her plump ruddy infant, is induced to sacrifice to the child of an opulent parent the natural food destined for her own offspring, which is then sent out to some hired nurse in some of the villages in the outskirts of the town, where good air, wholesome food, careful tending, and motherly attention are always promised, and sometimes faithfully bestowed. The event of this change is seen in the almost uniform history of the children of wet nurses, when banished from their mother's breast and their mother's care. The particular symptoms we shall presently give in the author's words, who has described them with accuracy and clearness. Neither is this evil confined to the children of wet-nurses, it is seen more or less in the young offspring of the lowest poor in this metropolis; and wherever the habitual intemperance and violent passions of the mother convert the naturally salubrious food for the infant into a noxious aliment; it is found in a greater or less degree, in a large proportion of the cases where infants are reared artificially from an early age, whatever care and attention are bestowed; and it is met with in infants even at the natural time of weaning, if from ignorance or inattention the great change of diet be not made with sufficient precaution to all the digestive functions.

We shall now give the author's description of the disease.

"The disease which I am now to consider, and which is the chief object of the present paper, is somewhat allied to the last in its nature, and is vulgarly denominated in this part of Scotland The Weaning Brash. It is one of the most fatal of the diseases of children, and, as far as I know, it is overlooked by those physicians who have made these diseases their study.

"It is an atrophy, the consequence of weaning children too suddenly at an unfavourable season of the year.

"This disease sometimes comes on two or three days after weaning; frequently not for three or four weeks; sometimes not before five or six weeks have elapsed.

"The

" The first symptom is a purglng, with griping pain, in which the dejections are usually of a green colour. When this purging is neglected, and, after continuing for some time, there is added a retching, with or without vomiting; when accompanied by vomiting, the matter brought up is frequently coloured with bile.

" These increased and painful actions of the alimentary canal, produce a loathing of every kind of food, and naturally are attended with emaciation and softness of the flesh, with restlessness, thirst, and fever.

" After some weeks I have often observed a hectic blush on the cheek; but the most characteristic symptom of this disease is a constant peevishness, the effect of unceasing griping pain, expressed by the whine of the child, but especially by the settled discontent of his features; and this expression of discontent is strengthened towards the conclusion of the disease, when the countenance has shared in the emaciation of the body.

" In the progress of the disease, the evacuations from the belly show very different actions of the intestines, and great changes in the biliary secretion; for they are sometimes of a natural colour, at other times slimy and ash coloured, and sometimes lienteric.

" Towards the end of the disease, the extremities swell, and the child becomes exceedingly drowsy; but these I rather conceive to arise from debility than to be pathognomic symptoms. It is remarkable, in the advanced stages of the disease, that the purging sometimes ceases for a day or two, but without any amelioration of the bad symptoms; nay, I think that children decay even faster than when the purging is most violent.

" The disease seldom proves fatal before the sixth or seventh week; and in this short time I have seen the finest children miserably wasted. I have seen, though rarely, a child recovered after the disease had continued three or four months; and again, I have seen the disease cut short by death, in the second, third, or fourth week, before it had reached the acme; the sudden termination having been occasioned by an incessant vomiting and purging, or by convulsions, from the immense irritation in the bowels.

" The disease is more frequent in children who have been weaned before the eighth or ninth month, and in particular, in those who, in consequence of some accident happening to the nurse, have been weaned abruptly.

" I have not been able to determine what temperament is most peculiarly liable to this disease; but, without meaning to insinuate any necessary relation, I think it appears most frequently in those children of a lax fibre, whose constitutions, at a more advanced stage of life, might be supposed liable to the attack of strumous disorders.

" This is a disease of the autumnal months. I seldom, comparatively speaking, have seen it commence before the soltice, nor after the end of the year; and I suspect that it is most general in sultry seasons.

" As

"As it will be presently shown, this disease gives origin to a great change in the glandular system of the mesentery; and this explains how it should happen, that after it has been removed, either by medicine, or by a proper regimen, and the healthful exertions of a good constitution, it is very apt, after slight errors in diet, or from cold, to return, even after the lapse of months. A person who knows this disease, will often be able to recognise it in the very obstinate and baffling complaints of the bowels, which children have from the beginning of the second to the end of the third year.

"At the time when weaning brash comes on, the teeth are usually appearing; and, from a common notion, that a flux is wholesome during teething, the disease is sometimes allowed to make an irremediable impression on the constitution, before the physician is called.

"My attention was very early directed to this disease, from finding that it had an appropriate name among the vulgar, and yet that it was not known to those physicians whom I consulted respecting its nature. Some of them had observed a purging as a very common consequence of weaning; but they supposed that it arose from teething: Others told me, that it arose from a mesenteric enlargement in scrophulous children: And until I could satisfy myself by dissection, I rested on this latter supposition."

The appearances on dissection are important. "The first dissection which I had an opportunity of making of a child who had died of weaning brash, did not instruct me in the true nature of the disease; for the mesenteric glands were considerably enlarged and inflamed, and I still imagined that their affection might have occasioned the purging and marasmus. But in prosecuting my research, I was convinced, that the disease was an undescribed one; and that although there might, in some instances, be mesenteric obstruction, it was not necessary to the disease; that it was the effect, and not the cause of it.

"I observed, in every instance, that the intestinal canal, from the stomach downward, abounded with singular contractions, and had in its course one or more intus-susceptions; that the liver was exceedingly firm, larger than natural, and of a bright red colour, and that the enlarged gall bladder contained a dark green bile. In some dissections, the mesenteric glands were swelled and inflamed; in others, however, they were scarcely enlarged, and had no appearance of inflammation.

"These contractions and intus-susceptions were entirely of a spasmodic nature, as in the latter the contained part of the gut was easily disengaged from that which formed its sac; and in no part of the entanglement was there adhesion, or even the mark of inflammation: and the contracted portions of the intestine were again permanently dilated, by pushing the finger into them.

"These appearances led me to imagine, that the weaning brash in its confirmed state, is imputable to an increased secretion of acrid crid bile, or rather to the morbid state of the liver, which occasions this; of which, however, I am afraid to attempt the explanation. It is proved, that there is an increased quantity of bile in the intestines, by the green dejections which are frequent in the beginning of the disease, and by the bilious vomiting."

As the cutting of teeth is an abundant source of irritation to many children, and of anxiety to all parents for great part of the first two years of the infant's life, it is natural that this should take its share in the supposed cause of the atrophy of weaned children. Nor is it easy (if possible) always to distinguish how far the symptoms of irritation may be produced, or at least aggravated, by the cutting of teeth. We think however that the author very justly lays but little stress on this cause in the present complaint; and it is of importance to determine this point as nearly as may be, since the practice of lancing the gums, we are convinced, is not quite so invariably harmless as is sometimes supposed.

The treatment of this very important disease is difficult, not so much as to what plan *ought to be prescribed*, as to that which *will be followed*.

The first and most obvious remedy would be to procure for the little sufferer the food for which it daily and hourly thirsts, *its* mother's or *a* mother's milk; but the very circumstances which at first deprived it of its natural food, will in most instances continue to operate in preventing its return to this aliment.

The food should be the most easily digestible, and in particular it should be remarked that animal food in the form of broth, jelly, &c. is much preferable to the vegetable, or at least should be largely mixed with it.

As to medicines, the author thus speaks of them: "Before I had formed the opinion of the disease which I now hold, I limited my attempts to the alleviating of the more urgent symptoms, endeavouring sometimes to restrain the purging by opiates, and at others anxious and happy to restore it again. I therefore used opiates in all ways, with aromatics; then the testaceous powders, with occasional doses of rhubarb. I tried laxatives in the beginning of the disease, and I think that they were useful. Then imagining the disease to be dysenteric, I gave ipecacuan, both as an emetic, and in small doses, mixed with prepared chalk, as an antispasmodic, to restrain the irregular action of the bowels, and certainly with some effect. Although I had some success from these remedies in the early stages of the disease, I found invariably, that when the disease had taken a firm root, it frustrated all my exertions.

"In the beginning of the disease, and even at all periods of it, when the attack is slight, I should certainly recommend a dose or two of rhubarb, to the extent of five or six grains, at the interval of two days between each dose; and that, in the mean time, the child should take half or a third part of a grain of ipecacuan powder, mixed with six or eight grains of prepared chalk, and a small portion of some aromatic powder, as cassia, every four or five five hours. Should there be much griping along with the purging, a glyster of mucilage of starch, with five or six drops of laudanum in it, administered at bed-time, will be attended with much advantage."

But the author speaks with more confidence of calomel, given as in other idiopathic diseases of the liver for a certain length of time, and more as a mercurial than a purgative. From the manner in which the author announces this remedy, he appears in some degree to consider it a practice as novel as it is beneficial. Of the salutary and often surprizing effects produced by this invaluable preparation in this disease, we have not a moment's doubt; but the concurring practice of the most skilful of the medical profession has so long been directed to this remedy, to the full as much in the maladies of children as in those of adults, that the testimony of our author to its value can now be only considered as an accession to a very large body of proof, and by no means as original and solitary evidence.

The volume concludes with twelve well-selected cases of this disease.

This Essay well deserves a perusal. The subject is peculiarly interesting, though it has not come within the author's plan to speak of its vast extent and the importance which attaches to it in a general point of view. Two observations we shall add, in proof of the peculiarly unfortunate situation in which (with some exceptions however) are placed the infants destined to the care of women who in their *own houses* receive them for hire; the one, that these miserable children are condemned to a most pernicious, because indiscriminate, use of opiates, in one form or other, to quiet their incessant cries of pain; and the other, that the active humanity of this metropolis has more than once been publicly directed to them, in a manner which shews the melancholy truth of all that has been represented concerning these half-abandoned infants.

---

*The Anatomist's Vade-Mecum, &c. by* Robert Hooper, M. D. *The Fifth Edition, to which are now added, Anatomical, Physiological, Medical, and Surgical Questions for Students.*

The rapid sale of this Compendium of Anatomy is, in this instance, a merited testimony to its value, and we only here notice the fifth edition, to mention the anatomical questions which make a small accession to the volume. They are 148 in number, and sufficiently well chosen, if it were an object to select just so many from the hundreds that might be asked with equal propriety. But these 148 questions, we are told, are "to be learnt by every student previous to his examination at Surgeon's Hall, the Medical Board, &c." a circumstance which attaches much more importance to them than they would otherwise merit. Does the author mean to assure the young candidate, that these will be the *only* questions asked? and that to obtain the testimonial of having been "*deliberately examined*

*and*

*and found fit and capable to exercise the art and science of surgery,"* the candidate may not be expected to inform the Worshipful Court of Examiners, how an hæmorrhage of an artery is to be stopped? How amputation is to be performed? How a fractured limb is to be disposed of? How a dislocation is to be reduced? None of which form a part of the 148 questions here set down. In fact, it is only in the Anatomy that the questions are equally full and select: the Surgery, Medicine, and even the Physiology, are highly defective; and in a future edition, we would advise that these be enlarged, and the plan of the questions, which is in itself useful, be confined to special objects, which set the mind of the answerer a reasoning, such as, "What is the cause of the bile regurgitating into the gall-bladder?" than merely such as "describe the heart—describe the uterus, &c."

